# Invest in physical activity to protect and promote health: the 2020 WHO guidelines on physical activity and sedentary behaviour

**DOI:** 10.1186/s12966-020-01051-1

**Published:** 2020-11-26

**Authors:** Hidde P. van der Ploeg, Fiona C. Bull

**Affiliations:** 1 Department of Public and Occupational Health, Amsterdam Public Health Research Institute, Amsterdam UMC, Vrije Universiteit Amsterdam, van der Boechorststraat 7, 1081BT Amsterdam, the Netherlands; 2 Sydney School of Public Health, The University of Sydney, Sydney, Australia; 3 Physical Activity Unit, Department of Health Promotion, World Health Organization, Geneva, Switzerland

## Abstract

In this editorial we discuss the new 2020 World Health Organization guidelines on physical activity and sedentary behaviour and a series of related papers that are published simultaneously in IJBNPA. The new guidelines reaffirm that physical activity is a ‘best buy’ for public health and should be used to support governments to increase investment in policy and research to promote and ensure physical activity opportunities are available for everyone. New recommendations on sedentary behaviour and inclusion of specific guidelines for people living with disability and/or chronic disease and pregnant and postpartum women are major developments since 2010. We discuss research priorities, as well as policy implementation and the contribution to the sustainable development agenda. The new guidelines can catalyse the paradigm shifts needed to enable equitable opportunities to be physically active for everyone, everywhere, every day.

To accompany the new 2020 *World Health Organization (WHO) guidelines on physical activity and sedentary behaviour* [1], the International Journal of Behavioral Nutrition and Physical Activity (IJBNPA) is publishing a series of papers providing more background and discussion of the guidelines and their implications for research and practice. These papers are published as part of a collaboration involving WHO, the Guideline Development Group (GDG), other scientists, the British Journal of Sports Medicine, the Journal of Physical Activity and Health, and IJBNPA.

The papers in this IJBNPA series focus on the new guidelines for children and adolescents aged 5–17 [2]; the sedentary behaviour recommendations for adults [3]; the role of physical activity in falls prevention [4] and prevention of osteoporosis [5], which were both commissioned reviews to inform the development of the WHO guidelines; and a paper on research priorities resulting from the guidelines development process [6]. An additional paper presents new analyses exploring the association between physical activity and income and gender inequality across the globe [7]. The commentary by Segar and colleagues discusses the implications of the new 2020 WHO guidelines for behavioural change communication [8]. In this editorial we describe the additions and changes in the new 2020 global guidelines and explore what actions are needed next, to ensure the opportunities created by these new global recommendations support everyone to be more active, everywhere, every day.

## Invest in physical activity – a ‘best buy’ for public health

The 2020 WHO global guidelines reaffirm that investment in physical activity continues to be a “best buy for public health”, as stated by Professor Jeremy Morris more than 25 years ago [9]. They reaffirm the importance of physical activity and provide an ever-growing list of health benefits that can lead to a reduction in burden of non-communicable disease (NCD) and improve daily functioning, mental health and wellbeing. A recent conservative estimate showed that 3.9 million death are already prevented annually because of a physically active lifestyle [10]. However, earlier estimates suggest the potential is even greater, and that an additional 5.3 million global deaths could be averted annually by supporting people to be more active [11]. Worldwide demographic changes, including ageing workforces and populations, as well as the COVID-19 pandemic, have made it clear there is a dire need for all countries to strengthen efforts in the prevention and management of chronic disease and invest in population-based prevention through public health systems and services.

Globally, the COVID-19 crisis and the resulting restrictions have exacerbated obesogenic environments for many people, where access to healthy foods and physical activity have become more difficult [12]. Although the exact impact is not known, COVID-19 has likely worsened the physical activity levels and caused a dramatic rise in sedentary behaviours in most countries. This combined syndemic of COVID-19 and NCDs [12] increases the urgency for governments at all levels and relevant stakeholders to invest in protecting and promoting policy action and research on physical activity.

## Too much sedentary behaviour is unhealthy

The new recommendation to reduce sedentary behaviour highlights the need to consider the full physical activity spectrum [3]. For individuals who are sedentary most of the time and do no to very little activity, it is particularly important to start to do some physical activity and limit sedentary time. Next to moderate and vigorous intensity physical activity, light intensity activity can play an important role in displacing high levels of sedentary behaviour and might be more feasible for some. This is especially relevant for people for whom the recommended 150–300 min/wk. of moderate to vigorous intensity physical activity might be challenging, such as people with impaired physical functioning. The guidelines emphasise that all types and intensities of activity count, bringing greater attention to the benefits and opportunity for everyone to be active in their own way, even if only of light intensity. This brings us to the other major development since the 2010 guidelines, namely the focus on inclusivity and new recommendations for specific subpopulations.

## Physical activity is good for everyone

The 2020 global guidelines aim to promote inclusivity through careful and purposeful language choice. For example, the recommendations do not refer to sedentary behaviour generically as “sitting”. Firstly, because there are some people living with a disability who can only sit or lie down, and secondly because it is possible to be active in a ‘sitting’ position. The guidelines' inclusivity agenda is most obviously seen in the development of the first global recommendations for specific key population groups, namely people living with disability, people living with chronic disease and for pregnant and postpartum women. Although the guidelines expose the clear need for more research in these populations, available evidence was considered and, when needed extrapolated from the general population, to inform these population specific recommendations. The launch of these specific guidelines emphasize that being physically active is important and beneficial for everyone, and in line with the UN’s Convention of the Rights of Persons with Disabilities opportunities to be physically active could be considered a basic human right [13]. However, guidelines are just the first policy step. They must now be used to strengthen and accelerate inclusivity in all physical activity policies and actions in order to close the inequities in opportunities for people of all ages and abilities to be regularly, safely and enjoyably active, especially those living with disability and chronic disease. This is a well overdue priority focus for researchers and policy makers alike.

## Advancing science and addressing evidence gaps

The process of developing the global guidelines identified key evidence gaps and these are summarised in the manuscript by DiPietro et al. providing clear direction on what research is needed to advance knowledge and inform future guidelines [6]. It is remarkable how often many of these gaps have been repeatedly highlighted over the past decade [14–18]. We must capitalise on the launch of the new 2020 global guidelines to engage the research community and research funders in more meaningful and effective collaboration to prioritise and invest in what evidence is most needed, not what studies are most amenable. Bold investment is needed to scale up research on physical activity and sedentary behaviours and in particular to strengthen research capacity in low resource contexts and low- and middle-income countries. This will not be easy, and notable challenges include the need to invest in studies with longer time frames than the typical one to three year cycles of many funding agencies as well as accelerating the use of wearable devices to strengthen measures of exposure. Increasing research on physical activity in specific populations, such as those living with disability and chronic disease, is needed and will require developing and nurturing multidisciplinary collaborations across clinical, preventive, and social care systems and services. Other areas highlighted by DiPietro and colleagues include the need for more high-quality research on the health benefits across the life course on the dose-response relationships between physical activity and sedentary behaviour, on light intensity physical activity, and on different types and domains of physical activity and sedentary behaviour.

## Creating a more active world

The 2020 global guidelines reaffirm the substantial health benefits afforded to all people from being more physically active and reducing sedentary behaviours. They reinforce the call outlined in the recent WHO Global Action Plan for Physical Activity (GAPPA) 2018–2030 [19], for a paradigm shift in how society views, plans for and supports physical activity. This shift can be achieved through coherent and sustained cross-government actions towards a ‘whole of system’ approach which aims to support everyone to be more active in ways they enjoy and in the places they live, work and play. Although the importance of physical activity in public health has gained increased recognition in global health policy in recent years, for example with global targets set for 2025 and 2030, there is still a long way to go. Only 78 out of 194 countries have national physical activity guidelines [20]. There is great need for all of us to fully engage in dissemination and support for country adoption and implementation.

Developing guidelines are an important stepping stone in the pathway of translating science into policy and as such are timely. Yet, real change will only come about when policy is translated into actual practice. GAPPA outlined the way forward providing all countries with a set of 20 recommended policy actions for governments and other stakeholders to adapt and tailor to local context [19]. Collectively these recommendations offer political and meaningful “win-wins” as implementation will contribute to achieving not only UN Sustainable Development Goal target 3.4, to reduce premature mortality from NCDs by one third through prevention and treatment and promote mental health and well-being by 2030, but will also contribute to achieving other goals such as healthy cities and communities, quality education, gender equality and mitigate climate change [21]. It is therefore opportune for everyone to use the policy window of the new 2020 WHO guidelines on physical activity and sedentary behaviour to escalate the case for investing in physical activity, in its own right, and as a multiplier of benefits towards better health and sustainable development as outlined in GAPPA [19].

Global advocacy for investment in physical activity will be the focus of the forthcoming WHO Global Status Report on Physical Activity in 2021 alongside continued development and dissemination of ACTIVE, a global set of practical toolkits for policy implementation across multiple settings, with a prioritised focus on low- and middle-income countries. As the COVID-19 pandemic has changed the world and challenged the status quo of so many aspects of how we live and work, let us all use the latest science and new 2020 global guidelines to ensure supporting people to be physically active is embedded in national and local recovery and development plans. There has never been a more important time for our collective voices and action.
